# Hyaluronic Acid in Bone Regeneration: Systematic Review and Meta-Analysis

**DOI:** 10.3390/dj12080263

**Published:** 2024-08-19

**Authors:** Claudia Lorenzi, Andrea Leggeri, Ilaria Cammarota, Paolo Carosi, Vincenzo Mazzetti, Claudio Arcuri

**Affiliations:** 1 Department of Clinical Science and Translational Medicine, University of Rome Tor Vergata, 00133 Rome, Italy; claudia.lorenzi@students.uniroma2.eu (C.L.); arcuri@med.uniroma2.it (C.A.); 2 Department of Chemical Science and Technologies, University of Rome Tor Vergata, 00133 Rome, Italy; andrea.leggeri@students.uniroma2.eu (A.L.); ilaria.cammarota@students.uniroma2.eu (I.C.); vincenzo.mazzetti@alumni.uniroma2.eu (V.M.)

**Keywords:** hyaluronic acid, bone regeneration, bone substitutes, systematic review, meta-analysis

## Abstract

Aim: The aim of this systematic review and meta-analysis was to assess possible histomorphometric differences in new bone formation and in remaining graft particles when hyaluronic acid (HA) was added and mixed with graft materials in bone regeneration. Materials and methods: This review was registered at the International Prospective Register of Systematic Reviews (PROSPERO) of the National Institute of Health Research (registration number CRD42024530030). Electronic research was performed, and involved studies published up to 29 February 2024 using a specific word combination. The primary outcome was to assess possible histomorphometric differences in new bone formation and in remaining graft particles when HA was added and mixed with graft materials in bone regeneration. The search resulted in 138 potential studies. Meta-analyses were performed using the fixed and random effects model to identify significant changes in new bone formation and in the remaining graft particles. Results: After screening procedures, only three randomized controlled trials fulfilled the inclusion criteria and were selected for qualitative and quantitative analysis. The effect size of HA in the new bone formation was not statistically significant at 95% CI (Z = 1.734, *p*-value = 0.083, 95 % CI -,399; 6516). The effect size of HA in the remaining graft particles was not statistically significant at 95% CI (Z = −1.042, *p*-value = 0.297, CI -,835; 255). Conclusions: Within the limitations of the present systematic review and meta-analysis, the addition of HA to bone graft did not result in significant changes in bone regeneration procedures in terms of new bone formation and residues, even if the included studies showed encouraging and promising results.

## 1. Introduction

Guided bone regeneration (GBR) can be considered one of the most documented techniques in the literature. It consists of inserting graft material, covered by a membrane, into bone defects to achieve adequate maintenance of the spaces and to enhance blood clot stabilization, osteoblast proliferation, and new bone formation [1]. Growth factors and cytokines released by the blood clot attract monocytes, macrophages, and osteochondroblast precursors, which enhance new blood vessel formation after bone regeneration [2].

Different graft materials have been used for bone regeneration, such as autologous bone, alloplastic, and xenoplastic materials [3]. Xenoplastic materials are the most commonly used due to their high availability and cost–benefit ratio. These materials offer stable bone volume maintenance, and several studies have shown that demineralized bovine bone material (DBBM) particles are osteoconductive and integrate well with newly formed bone [4]. Their long healing period and low percentage of new bone formation remain the main disadvantages of these graft materials [5]. In modern regenerative surgery, the use of graft materials with bioactive components is showing notable results as it induces activation of intracellular and extracellular molecular signaling pathways to promote tissue healing by accelerating the mechanisms of osteogenesis and improving the entire regenerative process [6]. One of the most commonly used bioactive components is hyaluronic acid (HA). HA is a high-molecular-weight polysaccharide that is an important component of the extracellular matrix (ECM) and is found in many different tissues of the human body [7]. HA is able to interact with proteoglycans and other bioactive molecules, is immunologically inert, and has a stimulating effect on angiogenesis [8]. HA maintains the viscoelasticity and the physical form of the ECM; it both supports its structure and functions as a lubricant [9].

It is widely recognized that the biological effects of HA are significantly influenced by its molecular size, with low-molecular-weight and high-molecular-weight HA typically inducing opposite effects [10,11]. Chen et al. (2016) provided biological evidence that high-molecular-weight HA enhances early mineralization of dental pulp cells through CD44 mediation [12].

Due to its physicochemical and biological properties, HA has been combined with various grafting materials in GBR. Combining xenograft granules with HA creates a putty bone grafting material that can improve surgical handling properties for the bone reconstruction of defects with demanding defect morphologies. Studies have shown that HA promotes osteogenesis [13]. HA improves bone growth by acting as a carrier for osteoinductive compounds [14] and promotes uniform distribution and greater density of the newly formed bone, thus altering the morphology of the scaffold and improving mineralization [15]. In the study of Kim JJ et al. (J Periodontal 2016) [16], the application of HA in extraction sockets with chronic pathology showed significantly increased bone formation compared to the control. The same group in a subsequent study (Kim JJ et al. J Periodontal 2019) [17] compared the effect of HA and BMP-2 on endodontic–periodontic lesions and observed alveolar bone overgrowth in both experimental groups.

The principal cell surface receptor for HA in the human body is CD44, a transmembrane glycoprotein distributed in various cells, such as osteocytes, where it is a sensitive marker of osteocytic differentiation [18] or cementoblasts and periodontal ligament cells (PDL) [19]. CD44 plays an important role in the proliferation and mineralization of PDL and, furthermore, shows how HA promotes cell viability, mineralization, and upregulation over a longer period of time, as well as the expression of mineralization-associated genes in cementoblasts [20,21].

Since it is a natural component of the ECM, when HA is combined with other materials, it makes the surfaces of the graft biomimetic, promoting mesenchymal migration [22], adhesion, proliferation, and differentiation [23]. The use of HA combined with bone graft materials also offers advantages due to its antimicrobial properties on pathogens such as staphylococcus, streptococcus, Pseudomonas aeruginosa, Enterococcus, and S. mutants [24].

The aim of this systematic review and meta-analysis was to assess possible histomorphometric differences in new bone formation and in the remaining graft particles when HA was added and mixed with graft materials in bone regeneration.

## 2. Materials and Methods

### 2.1. Protocol and Registration

This review was registered at the International Prospective Register of Systematic Reviews (PROPERO) of the National Institute of Health Research (registration number CRD42024530030). This review was reported according to the “Preferred Reporting Items for Systematic Reviews and Meta-Analysis (PRISMA)” guidelines (Page et al. 2021) [25].

### 2.2. Population, Intervention, Comparison, Outcomes, and Study Design

The PICOS (Population, Intervention, Comparison, Outcomes, and Study design) format was used to establish the research question: “can biomaterials alone in regenerative surgery procedures (I), in partially edentulous patients (P), have the same histomorphometric results (O), in randomized clinical trials (S), as the use of biomaterials in association with HA (C)?”.

### 2.3. Inclusion and Exclusion Criteria

Before the start of the study, the inclusion and exclusion criteria were identified. To be included in the review, the article had to be written in the English language, a randomized clinical trial in humans, and had to contain the results of histomorphometric analyses of new bones. Systematic reviews, nonrandomized studies, commentaries, letters to the editor, in vitro studies, studies in animal models, studies where HA was not mixed with biomaterial, and studies that did not report histomorphometric results of the new bone in the research protocol were excluded.

### 2.4. Types of Intervention

The analyzed studies were randomized clinical trials or split-mouth randomized clinical trials in humans. The test group was treated with grafting materials in association with HA, and the control group was treated with grafting materials alone for bone regeneration.

### 2.5. Outcome Measures

The primary outcome of this systematic review and meta-analysis was to assess possible histomorphometric differences in new bone formation and in the remaining graft particles when HA was added and mixed with graft materials in bone regeneration.

### 2.6. Search Strategy

Electronic research was performed with MEDLINE databases and involved studies published up to 29 February 2024. The following combination of words were used: “hyaluronic acid and bone regeneration and dentistry”. In addition, bibliographies of the included articles were analyzed and cross-checked.

### 2.7. Selection Criteria and Data Analysis

Full-text screening, study selection, and data extraction were performed in duplicate, and disagreements were resolved by consensus. The analyzed studies were randomized clinical trials on human subjects assessing histomorphometric changes in sites requiring bone regeneration after treatment with DBBM or xenograft with or without the use of hyaluronic acid.

### 2.8. Risk of Bias

The quality of the included studies was assessed independently by the authors by means of Cochrane Collaboration’s tool 2. This tool was used to assess any potential risk of bias in the included RCTs [26]. The possible outcomes were a study with a low risk of bias, which provided all the required information about the investigated parameters; a study with a moderate risk of bias, which did not provide all the information required to fulfill the review process; and a study with a high risk of bias, which was missing more than 2 parameters.

### 2.9. Statistical Analysis

Meta-analyses were performed using the fixed and random effects model to identify significant changes in new bone formation and in the remaining graft particles. The Egger test, Cochran’s Q-statistic, and the I2 statistic were used to assess any publication bias and to calculate heterogeneity between the included studies. *p*-values < 0.05 were considered statistically significant. I2 values of 25%, 50%, and 75% corresponded to the cutoff points for low, moderate, and high degrees of heterogeneity, respectively. A minimum of 3 studies were needed to perform a meta-analysis.

## 3. Results

### 3.1. Included Studies

**Figure 1:**
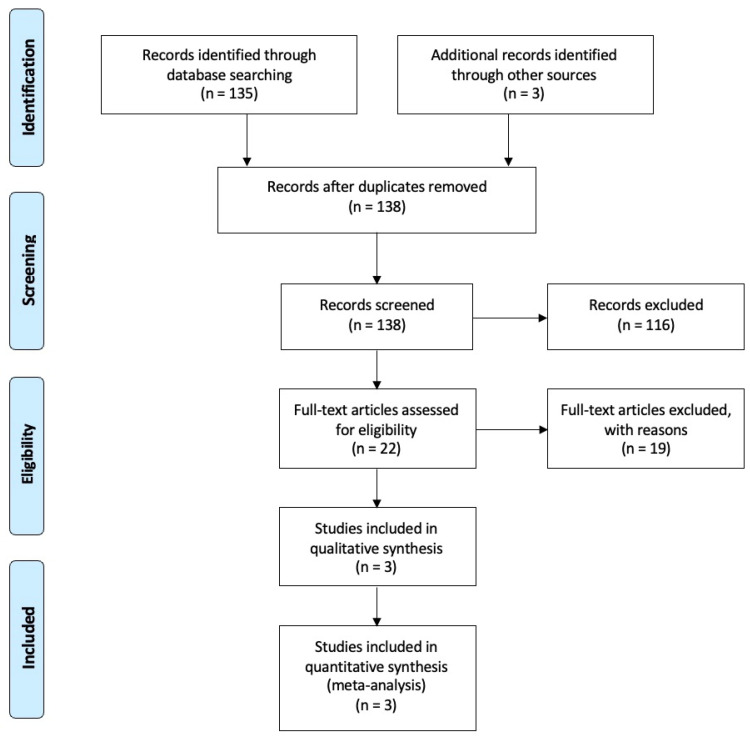
PRISMA (Preferred Reporting Items for Systematic Review and Meta-Analyses) search strategy flow chart.

The search strategy resulted in 138 articles. After examining the title and abstract, 116 were excluded. Of the 22 potentially relevant articles, the full text was examined, and 3 articles were included in the analysis (Figure 1).

The three articles included in the review are presented in Table 1.

### 3.2. Excluded Studies

Nine articles among those examined dealt with periodontal surgery [30,31,32,33,34,35,36,37,38] and one with peri-implantitis [39]. Two articles were reviews [40,41], and one study was not randomized [42]. In two studies, HA was not mixed with biomaterials [43,44], and three other studies were excluded because there were no histomorphometric data [45,46,47]. One article was excluded because the standard deviation was not present in the histological results data [48]. All excluded articles are summarized in Table 2.

### 3.3. Study Characteristics

**Figure 2:**
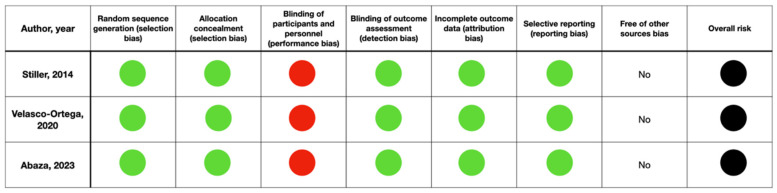
Quality assessment of the included studies following Cochrane Collaboration’s tool for assessing risk of bias in randomized trials. Green = low risk; red = high risk [27,28,29].

All selected studies were RCTs and published between 2014 and 2023. The risk of bias assessment reported a low risk of bias for the included studies (Figure 2).

One study was designed as split-mouth [27], evaluating the effect of two bone graft materials (pure, synthetic b-TCP granules with a grain size of 700e1400 mm and a putty material composed of pure, synthetic b-TCP granules with two types of grain size ranges, 125e250 mm and 500e700 mm, embedded in a sodium HA hydrogel matrix with a b-TCP: HA ratio of 10:1) on bone formation, bone matrix maturation, and osteoblast differentiation six months after maxillary sinus augmentation (MSA). In the study published by Velasco-Ortega et al. [28], the aim was to evaluate and compare, histomorphometrically and clinically, three different bone substitutes in the MSA. The studied materials were an organic bovine bone mineral (ABBM) and betatricalcium phosphate (TCP) with or without the addition of HA. A CBCT was performed before surgery and 9 months after the MSA before the implant surgery, where bone biopsies were performed for histomorphometric analyses. The last included study [29] involved patients who required implant placement in the upper arch. Patients were randomly divided into three groups (*n* = 12 each) receiving different treatments: injectable platelet-rich fibrin (I-PRF) with xenografts, HA with xenografts, or xenografts alone. Histomorphometric analysis was performed to determine the percentages of newly formed bone, mature bone, and residual grafts after 4 months (Table 3).

### 3.4. Included Studies’ Heterogeneity

**Figure 3:**
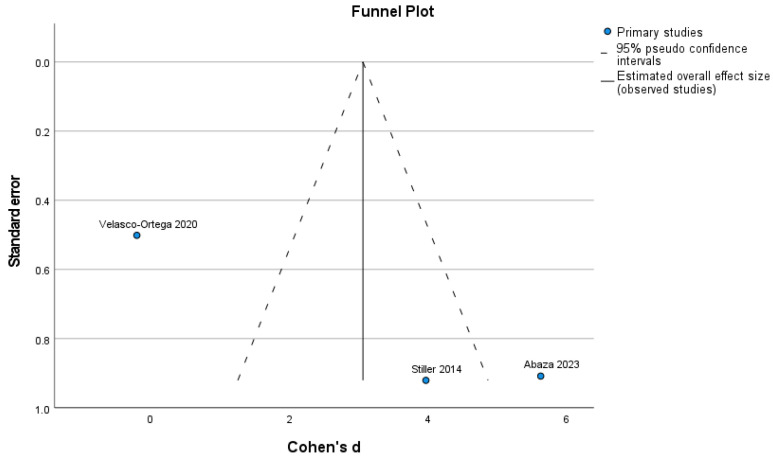
Funnel plot for new bone formation [27,28,29].

**Figure 4:**
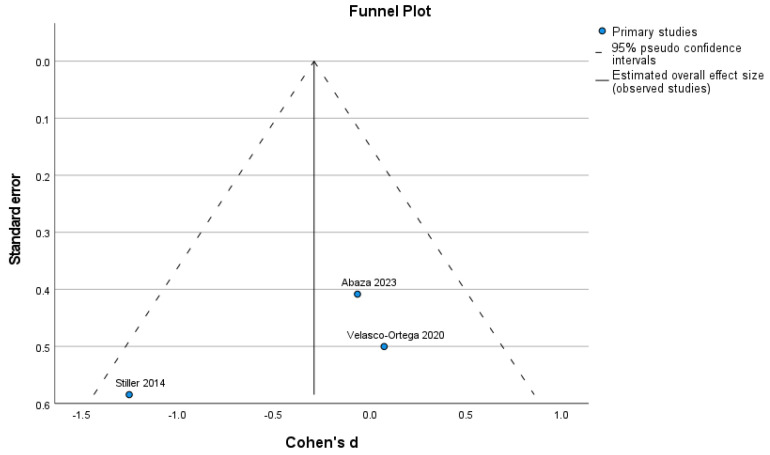
Funnel plot for remaining graft particle [27,28,29].

The Egger test was not statistically significant for any of the investigated outcomes, the new bone formation, with or without HA (*p* = 0.244), and the remaining graft particles (*p* = 0.427). The test for heterogeneity revealed a Cochran’s Q-statistic index of 39.104 (new bone formation) and 3546 (remaining graft particles). The I2 statistic indexes were 93.7% (new bone) and 41.4% (remaining graft particles) (Figure 3 and Figure 4).

### 3.5. New Bone Formation

**Figure 5:**
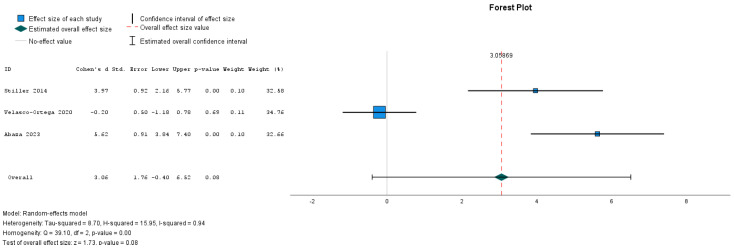
Forest plot for new bone formation [27,28,29].

The effect size of HA in new bone formation was not statistically significant at 95% CI (Z = 1.734, *p*-value = 0.083, CI-,399; 6516) (Figure 5).

### 3.6. Remaining Graft Particles

**Figure 6:**
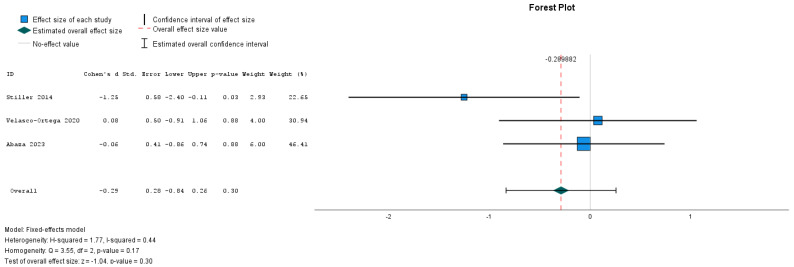
Forest plot for remaining graft particles [27,28,29].

The effect size of HA in the remaining graft particles was not statistically significant at 95% CI (Z = −1.042, *p*-value = 0.297, CI -,835; 255) (Figure 6).

## 4. Discussion

The aim of this systematic review and meta-analysis was to assess possible histomorphometric differences in new bone formation and in remaining graft particles when HA was added and mixed with graft materials in bone regeneration.

To the best of the authors’ knowledge, this is the first systematic review and meta-analysis assessing the potential effect of HA on bone regeneration procedures.

The main limitation of the present study was related to the paucity of well-conducted RCTs, which may be related to the novelty of the treatment. Nevertheless, three RCTs that assessed low sources of potential bias were included in the meta-analysis. Meta-analysis showed that the effect sizes of HA in new bone formation and in the remaining graft particles were not statistically significant at 95% CI (*p*-value = 0.083, CI -,399; 6516 and *p*-value = 0.297, CI -,835; 255).

After tooth loss, postextraction socket healing involves dimensional changes with loss of up to 50% of the original dimension [49], both in horizontal and vertical directions, especially on the buccal walls [50]. During this healing period, which lasts up to 12 months, the alveolar ridge can lose up to 5–7 mm [51]. Regenerative methods using growth factors, such as recombinant human platelet-derived growth factor-BB (rhPDGF-BB) and recombinant human bone morphogenetic proteins (rhBMPs), have been suggested. These techniques provide distinct benefits compared to traditional invasive surgical procedures for treating significant hard and soft tissue defects. The review by Galarraga-Vinueza et al. (2023) revealed that histomorphometric analyses have shown that rhBMP-2 significantly enhances new bone formation, increases bone marrow growth, and improves bone vascularity in grafted areas. Therefore, employing rhBMPs in bone grafting procedures can be advantageous for patients with impaired bone healing capacity, limited donor sites, a need for accelerated healing, or extensive bone defects. Additionally, numerous studies endorse the use of rhPDGF-BB for periodontal regeneration and root coverage procedures [52].

Since it is necessary to have an adequate bone volume and quantity of keratinized tissue to replace a lost tooth with an implant-supported crown in order to obtain good functional and esthetic results [53], in modern dentistry, it is imperative to anticipate the risk of hard and soft tissue loss and maintain adequate bone volume, especially in esthetic regions. It has been shown that inserting graft materials into the postextraction socket and covering them with a membrane to stabilize the graft and the clot and to avoid invasion of the soft tissues can prevent bone deficits and decrease physiological resorption [54,55]. This technique is called alveolar ridge preservation (ARP). In a 2023 split-mouth study [45], DBBM was used for ARP with and without HA in seven patients. Four months post-operatively, before implant placement, a Cone Beam Computed Tomography (CBCT) scan was performed. The results revealed that the combination of hydroxyapatite (HA) mixed with deproteinized bovine bone mineral (DBBM) exhibited significantly lower volumetric resorption. Additionally, the combination of DBBM with HA was associated with a greater level of homogenous incorporation of DBBM graft particles into the newly formed bone throughout the entire biopsy [45]. In another 2018 split-mouth study on ARP [46], 32 lower premolars were extracted from 16 patients. Sixteen sockets were filled with 1% hydroxyapatite (HA), while the other sixteen sockets were filled with a clot. Morphometric evaluation and fractal dimension analyses were conducted 30 and 90 days postsurgery using CBCT. Bone formation was more advanced in the sockets filled with HA after 30 days. However, after 90 days, both treated and control sockets exhibited similar bone density, suggesting that the biomaterial positively influenced the initial stages of alveolar healing. The test group filled with 1% HA showed higher levels of newly formed bone compared to the control group after 30 days, but no significant differences were observed after 90 days [46].

A previous meta-analysis by Domic et al. (2023) suggested that applying HyA could positively impact soft tissue healing following the nonsurgical extraction of normally erupted teeth. However, it appears that HyA does not significantly affect post-extraction alveolar ridge remodeling despite preclinical studies suggesting its potential to enhance bone formation [56].

In the posterior zone of the maxillary bone, pneumatization of the maxillary sinus can make implant rehabilitation difficult due to the lack of residual bone volume [57]. MSA is one of the best documented and most predictable bone regeneration techniques, with long-term implant success that is comparable to that achieved in native bone. The aim of this technique is to increase the bone volume in the posterior maxilla in order to place dental implants. In addition to an adequate volume, this should also guarantee correct bone quality to obtain immediate and long-term stability of the implants. The biomaterials used represent a k-factor in determining bone quality. Generally, two types of materials are used in MSA: autologous bone or bone substitutes, such as alloplastic or xenoplastic materials. Using bone substitutes simplifies the MSA procedure, as it avoids a second surgical site for bone harvesting and eliminates the risk of donor site morbidity [58,59]. In a previously published split-mouth study [43], 10 patients were treated with bilateral sinus floor elevation surgery using two techniques: hyaluronic acid and ultrasonic resorbable pin fixation (URPF) without any type of graft. CBCT was used to measure bone height at the sinus zone before and after 6 months of sinus floor elevation surgery. The idea was to use hydroxyapatite (HA) as a scaffold to provide optimal spacing and enhance vascular and cellular invasion. However, significant differences were observed in the height of the alveolar bone and the reduction in sinus volume on the URPF side [43].

## 5. Conclusions

Within the limitations of the present systematic review and meta-analysis, the addition of HA to the bone graft did not report significant changes in bone regeneration procedures in terms of new bone formation and residues, even if the included studies showed encouraging and promising results. The use of HA in bone regeneration may be useful in the future to stabilize the blood clot and enhance wound healing. However, more randomized clinical trials are needed to confirm such positive outcomes.

## Figures and Tables

**Table 1:** Main charachteristics of the included studies.

Study	Study Design	Number of HA Cases	% New Bone HA	% Particles HA	Number of Control Cases	% New Bone Control	% Particles Control
Stiller et al., 2014 [27]	Randomized split-mouth	7	30.1	29.5	7	17.4	32.9
Velasco-Ortega et al., 2020 [28]	Randomized controlled trial	8	23.29	7.17	8	23.85	7.17
Abaza et al., 2023 [29]	Randomized controlled trial	12	56.66	2.63	12	24.05	2.71

**Table 2:** Ecluded studies and reason for exclusion.

Studies	Exclusion Reason
Ballini et al., 2009 [30] Božić et al., 2021 [31] de Santana et al., 2015 [32] Mamajiwala et al., 2021 [33] Sehdev et al., 2016 [34] Engström et al., 2001 [35] Briguglio et al., 2013 [36] Pilloni et al., 2021 [37] Vanden Bogaerde et al., [38]	The focus of these studies is periodontal surgery
Kaya et al., 2019 [39]	The main focus was not bone regeneration
D’Albis et al., 2022 [40] Ostos-Aguilar et al., 2023 [41] Lorenz et al., 2018 [42]	Study design different from RCTs
Göçmen et al., 2016 [43] Eeckhout et al., 2022 [44]	HA was not mixed with biomaterials
Husseini et al., 2023 [45] Alcântara et al., 2018 [46] Baldini et al., 2010 [47]	No histomorphometric data were reported
Kauffmann et al., 2023 [48]	Missing statistical data to be included in meta-analysis

**Table 3:** Detailed protocol of the included studies.

Study	Bone Graft Materials	Purpose of the Study	Study Protocol	Histomorphometric Results
Stiller et al., 2014 [27]	TCP-G: CEROS TCP Granules, Mathys Ltd., Switzerland. Pure, synthetic b-TCP granules with a grain size of 700–1400 mm. TCP-P: CEROS TCP Putty, Mathys Ltd., Switzerland. Putty material composed of pure, synthetic b-TCP granules with two types of grain size ranges, i.e., 125–250 mm and 500–700 mm, embedded in a sodium HA hydrogel matrix with a b-TCP:HA ratio of 10:1.	Evaluate the effect of these two bone graft materials on bone formation, bone matrix maturation and osteoblast differentiation six months after MSA.	CBCT was performed preoperatively, post-operatively, and six months after MSA for a 3D assessment of the sinus floor anatomy and bone volume. Before the implant surgery, bone biopsies were performed for histomorphometric analyses.	Six months after SFA: TCP-G: Bone: 17.4 ± 3.3%, Particle: 32.9 ± 2.4% Marrow spaces: 49.7 ± 2.6%. TCP-P: Bone: 30.1 ± 3.1% Particle: 29.5 ± 3.0% Marrow spaces: 40.5 ± 3.2%
Velasco-Ortega et al., 2020 [28]	Control Group: Bio-Oss Cancellous, Geistlich, Wolhusen, Switzerland. Demineralized Bovine Bone Mineral Test group: Hyadent BG, Regedent. TCP in the test group plus crosslinked HA with a ratio of 2:1.	Evaluate and compare, histomorphometrically and clinically, different bone substitutes in the MSA.	A CBCT was performed before surgery and 9 months after the MSA before the implant surgery, where bone biopsies were performed for histomorphometric analyses.	Control Group: New bone: 25.97 ± 2.79% Particle: 32.19 ± 1.52% Marrow spaces: 41.99 ± 3.44% Test: New bone: 23.29 ± 2.01% Particle: 7.47 ± 3.59% Marrow spaces: 69.80 ± 2.51%
Abaza et al., 2023 [29]	Group 1: crosslinked HA solution (Perfecta) + cerabone^®^, Straumann, Germany. Group 2: cerabone^®^, Straumann, Germany.	Compare the effectiveness of HA in combination with xenografts for ARP versus xenografts alone.	Cone beam CT scans were performed preoperatively and 4 months post-operatively to measure radiographic bone gain. Histological assessment of core bone biopsies was performed 4 months post-operatively.	Group 1: New bone: 56.66 ± 7.35% (Mature bone: 18.26 ± 4.44%) Particle: 2.63 ± 1.27% Group 2: New bone: 24.05 ± 3.64% (Mature bone: 2.41 ± 1.36%) Particle: 2.71 ± 1.24%

## Data Availability

Data are available upon reasonable request from the corresponding author.
